# Factors Associated with Active Smoking, Quitting, and Secondhand Smoke Exposure among Pregnant Women in Greece

**DOI:** 10.2188/jea.JE20090156

**Published:** 2010-09-05

**Authors:** Constantine I. Vardavas, Evridiki Patelarou, Leda Chatzi, Theano Roumeliotaki, Katerina Sarri, Sharon Murphy, Antonis Koutis, Anthony G. Kafatos, Manolis Kogevinas

**Affiliations:** 1 Department of Social Medicine, Faculty of Medicine, University of Crete, Heraklion, Crete, Greece; 2 University of Minnesota, United States; 3 Center for Research in Environmental Epidemiology (CREAL), Barcelona, Spain; 4 Municipal Institute of Medical Research (IMIM-Hospital del Mar), Barcelona, Spain; 5 CIBER Epidemiologia y Salud Pública (CIBERESP), Spain

**Keywords:** smoking, cessation, pregnancy, fetal health, passive smoking, SHS

## Abstract

**Background:**

Pregnant women are exposed to tobacco smoke through active smoking and contact with secondhand smoke (SHS), and these exposures have a significant impact on public health. We investigated the factors that mediate active smoking, successful quitting, and SHS exposure among pregnant women in Crete, Greece.

**Methods:**

Using a cotinine-validated questionnaire, data were collected on active smoking and exposure to secondhand smoke from 1291 women who had successfully completed the first contact questionnaire of the prospective mother-child cohort (Rhea) in Crete during the 12th week of pregnancy.

**Results:**

Active smoking at some time during pregnancy was reported by 36% of respondents, and 17% were current smokers at week 12 of gestation. Those less likely to quit smoking during pregnancy were those married to a smoker (OR, 1.76; *P* = 0.008), those who were multiparous (1.72; *P* = 0.011), and those with young husbands. Of the 832 (64%) nonsmokers, almost all (94%, *n* = 780) were exposed to SHS, with the majority exposed at home (72%) or in a public place (64%). Less educated women and younger women were exposed more often than their better educated and older peers (*P* < 0.001). Adjusting for potential confounders, parental level of education, age, and ethnicity were the main mediators of exposure to SHS during pregnancy.

**Conclusions:**

Active smoking and exposure to SHS are very prevalent among pregnant women in Greece. The above findings indicate the need for support of population-based educational interventions aimed at smoking cessation in both parents, as well as of the importance of establishing smoke-free environments in both private and public places.

## INTRODUCTION

Tobacco consumption is a leading cause of preventable death.^1^ Tobacco contains a plethora of carcinogenic and volatile chemicals, and both active smoking and exposure to secondhand smoke (SHS) are related to the development of cancer and cardiovascular disease.^2^ Exposure to cigarette smoke in utero, whether via maternal smoking or maternal exposure to SHS, is associated with a number of adverse pregnancy outcomes,^3^ is a source of severe oxidative stress in the unborn child,^4^ and can have detrimental effects on fetal growth, neurodevelopment,^5^ neurobehavior,^6^ and cardiovascular regulation^7^ that predispose the fetus to a number of adverse health outcomes.^8^

Pregnancy is perceived by many to be a unique window of opportunity for smoking cessation, as motivation to care for the unborn fetus is typically high.^9^ Smoking cessation during pregnancy is also a significant method of harm reduction, as cessation early during pregnancy has been found to reduce the risk of infant death^10^ and prevent adverse pregnancy outcomes such as low birth weight, small for gestational age, and preterm delivery.^11^^,^^12^ Moreover, as cessation can subsequently influence the child’s exposure to tobacco smoke during infancy, it is important to identify the factors that mediate spontaneous cessation during pregnancy. To date, a number of factors have been found to influence the continuation of smoking during pregnancy, including parity, level of education, socioeconomic status, household SHS exposure, and the smoking habits of both parents. Psychosocial pressures, such as anxiety, high job strain, and exposure to violence, are also important.^13^^–^^17^ The factors that influence the continuation of smoking or maternal quitting attempts should be identified at a population level and acknowledged when designing interventional studies.

Because smoking prevalence among women in Greece is notoriously high,^18^ the purpose of this research was to investigate the factors that influence active smoking and maternal quitting during early pregnancy, and to identify the main determinants of exposure to SHS, among pregnant women in Crete, Greece.

## METHODS

### Aim of the Rhea study

The aim of the mother-child cohort in Crete (Rhea study) is to evaluate nutritional, environmental, biological, and psychosocial exposures in the prenatal period and early childhood and to determine how these are associated with the development of the fetus and child, and with the occurrence of chronic disease. Moreover, within the Rhea cohort, the FETAL (“Fetal and infant exposure to secondhand smoke and its role in acute and chronic disease development”) substudy was conducted to identify the extent to which unborn children in Greece are exposed to active maternal smoking and secondhand smoke, and to clarify how this exposure may predispose the unborn child to development of acute and chronic disease.

### Design of the study

The Rhea study examined a mother-child cohort of pregnant women (Greeks and immigrants) who were residents of the prefecture of Heraklion, Crete. All women who became pregnant during the 1-year period from February 2007 until February 2008 were contacted and asked to participate in the study. The first contact took place around week 12 of gestation, in the form of a face-to-face, computer-assisted, interview. The study was approved by the relevant ethical committee, and all participants provided written informed consent. During the study period, 2221 women were contacted, of which 461 did not meet the entry criteria (ie, they had a limited understanding of Greek or were nonresidents of the prefecture of Heraklion) and 154 refused to participate, leaving a study population of 1606 pregnant women who agreed to participate. The data presented in this manuscript refer to cross-sectional information collected during the first major interview (which took place in approximately the third month of pregnancy) of the 1291 women from which complete questionnaire data were available.

### Smoking and SHS exposure

Information on both maternal and paternal sociodemographic characteristics was collected, as was extensive information on both parents’ self-reported smoking habits and exposure to SHS. Smoking status was classified into 3 categories. Nonsmokers were classified as women who reported not smoking for at least 3 months before pregnancy; ex-smokers were classified as those who reported smoking some time within the 3 months before pregnancy or some time during the first 12 weeks, but who had quit since; and active smokers were classified as those who reported smoking 3 months before pregnancy, during early pregnancy, and at the time of interview, which took place at approximately week 12 of gestation. Information on maternal exposure to SHS was also collected. Specifically, mothers were asked to report if they had any exposure to SHS during their pregnancy and from which source. The 5 different sources that were investigated were the family home, the mother’s workplace, cars, public venues (restaurants, cafés), and miscellaneous sources, such other homes, social gatherings, etc. In addition, the mother’s cumulative number of SHS exposure sources was determined by adding the number of different sources to which she was exposed (house + work + car + public venues + other places).

### Questionnaire validity

To evaluate the validity of the SHS questionnaire, urine samples were collected from 33 randomly selected nonsmoking women. The samples were analyzed for total cotinine (cotinine plus cotinine *N*-glucuronide) by treating them with a base to cleave the glucuronide conjugates before gas chromatography–mass spectrometry analysis, as previously described.^19^ Cotinine levels were log-transformed due to the skewness of the distribution, and the log cotinine levels were compared to self-reported levels. Those exposed to SHS in the house had approximately 3 times the geometric mean cotinine levels of those who had not been exposed (14.4 ng/ml vs. 4.4 ng/ml, *P* < 0.02). Similarly, those who reported exposure to SHS in public places had almost 3 times the level of those who did not (18.2 ng/ml vs. 6.9 ng/ml, *P* < 0.02).

### Statistical analysis

Descriptive data are presented as mean ± standard deviation for continuous variables and as percentages (*n*) for categorical variables. Bivariate associations between dependent and independent variables were evaluated using Pearson’s chi-square test for categorical variables and the *t*-test for continuous variables. All hypothesis testing was conducted assuming a 0.05 significance level and a 2-sided alternative hypothesis. Multivariable logistic regression models were further applied to investigate the factors that may mediate smoking before pregnancy, quitting during pregnancy, and maternal exposure to SHS during pregnancy. The statistical package SPSS 16.0 was used to perform the analysis.

## RESULTS

### Smoking and quitting among pregnant women in Greece

The prevalence of any smoking during pregnancy was estimated at 36% of all pregnant women, among which 19% quit, leaving 17% of pregnant women as active smokers during the 12th week of pregnancy (Table 1). The participants’ educational level was strongly associated with maternal smoking status: only 13% (*n* = 47) of higher-educated mothers were active smokers, as compared with 25% (*n* = 66) of lower-educated mothers (*P* < 0.001). Further, the percentage of smokers among women married to higher-educated husbands was lower than that among those married to lower-educated husbands (12% vs. 24%, *P* < 0.001). In addition, both the husband’s and the pregnant mother’s ethnicity were significant determinants of smoking status during pregnancy.

**Table 1.** Smoking status^a^ and sociodemographic characteristics of pregnant women and their husbands in the Greek RHEA birth cohort study, 2007–2008

		Nonsmoker	Ex-smoker	Active smoker	*P* value^d^
Age^b^	Maternal age (mean ± SD)	29.7 ± 5.1	28.9 ± 4.9	29.1 ± 5.3	0.063
	Paternal age (mean ± SD)	33.5 ± 5.8	32.5 ± 5.7	32.2 ± 5.5	**0.001**

Residence^c^	Urban % (*n*)	64 (606)	18 (168)	19 (180)	0.210
	Rural % (*n*)	68 (193)	14 (38)	18 (51)	

Ethnicity^c^	Greek % (*n*)	63 (734)	18 (207)	19 (222)	**0.004**
	Non-Greek % (*n*)	77 (98)	8 (10)	16 (20)	

Paternal ethnicity^c^	Greek % (*n*)	63 (753)	18 (211)	20 (238)	**<0.001**
	Non-Greek % (*n*)	80 (90)	4 (5)	16 (18)	

Education^c^	Low % (*n*)	62 (167)	13 (35)	25 (66)	**0.001**
	Medium % (*n*)	61 (394)	20 (127)	19 (122)	
	High % (*n*)	72 (264)	15 (55)	13 (47)	

Paternal education^c^	Low % (*n*)	62 (286)	15 (67)	24 (110)	**<0.001**
	Medium % (*n*)	65 (345)	19 (101)	17 (88)	
	High % (*n*)	70 (184)	18 (47)	12 (32)	

Parity^c^	Multipara % (*n*)	64 (509)	15 (121)	21 (164)	**0.024**
	Primipara % (*n*)	65 (319)	19 (95)	16 (76)	

Total	Smoking status % (*n*)	64 (832)	19 (242)	17 (217)	n/a

^a^Current smoking defined as a pregnant woman self-reported as a smoker at the 12th week of gestation.

^b^2-sided *t*-test.

^c^2-sided chi-square test.

^d^*P* values based on 2-sided tests, with values <0.05 regarded as statistically significant.

We also examined factors associated with smoking before pregnancy (active smokers and ex-smokers vs. nonsmokers) in a regression model adjusted for potential confounders, as described in the Methods (Table 2). Active smoking before pregnancy was related to having a husband who smoked (OR, 2.90; 95% CI, 2.25–3.75) and to low maternal education (1.40; 1.06–1.85). Specifically, lower-educated women were more likely to be smokers than were higher-educated mothers (1.40; 1.06–1.85). A protective effect was observed for being married to a non-Greek husband (0.41; 0.23–0.74), for living in a rural area (0.68; 0.49–0.92), and for every year of increase in her husband’s age (0.96; 0.95–0.97).

**Table 2.** Factors associated with smoking before pregnancy and quitting during pregnancy of the study population in the Greek RHEA birth cohort study, 2007–2008

		OR^a^	95% CI	*P* value^b^
**Factors associated with smoking before pregnancy** **(Current/ex-smokers vs. nonsmokers)**				
Paternal age	*Change per year*	0.96	0.95–0.97	<0.001
Paternal ethnicity	*Greek*	1.00		
	*Non-Greek*	0.41	0.23–0.74	0.003
Education level	*High*	1.00		
	*Mid/low*	1.40	1.06–1.85	0.018
Residence	*Urban*	1.00		
	*Rural*	0.68	0.49–0.92	0.013
Paternal smoking status	*Nonsmoker*	1.00		
	*Smoker*	2.90	2.25–3.75	<0.001

**Factors associated with continued smoking during pregnancy** **(Current vs. ex-smokers)**				
Paternal age	*Change per year*	0.98	0.97–0.99	0.002
Paternal ethnicity	*Greek*	1.00		
	*Non-Greek*	3.03	0.95–9.70	0.062
Paternal smoking status	*Nonsmoker*	1.00		
	*Smoker*	1.76	1.16–2.67	0.008
Parity	*Primipara*	1.00		
	*Multipara*	1.72	1.13–2.61	0.011

^a^Backward logistic regression analysis was conducted with the following factors in step 1: maternal and paternal age, level of education (high vs. mid/low), ethnicity (Greek vs. immigrant), paternal smoking status, parity (primipara vs. multipara), place of residence (urban vs. rural), and maternal occupational status (working vs. unemployed or on leave). In the first regression analysis an OR >1 indicates women more likely to be smokers; in the second regression analysis, an OR >1 indicates women who continued to smoke during pregnancy and did not quit.

^b^*P* values based on 2-sided tests, with variables excluded when *P* > 0.1. Statistical significance was defined as *P* < 0.05.

The factors that influenced maternal quitting during pregnancy were mostly related to paternal characteristics (Table 2). Women married to smokers (1.76; 1.16–2.67) and to non-Greek husbands (3.03; 0.95–9.70), were less likely to quit during pregnancy, while the husband’s age had a protective effect (0.98; 0.97–0.99, per 1 year of increase). Additionally, multiparous women were also less likely to quit (1.72; 1.13–2.61).

### Exposure to SHS among pregnant women in Greece

As shown in Table 3, a large percentage of pregnant women had been exposed to SHS, with 72% exposed to SHS at home, 64% in public places, and 49% at work. Regarding household exposure to SHS from at least 1 family member, 84% of the lower-educated mothers were exposed to household SHS, in comparison with only 58% of the higher-educated mothers (*P* < 0.001), while only 48% of those living with higher-educated husbands were exposed to SHS, in comparison with 84% of those married to lower-educated husbands (*P* < 0.001). Furthermore, the number of children in the household was also found to mediate maternal exposure to SHS: mothers were exposed to SHS in 79% of households with 3 or more children, as compared with 67% of households with 1 or 2 children (*P* = 0.007). Husbands were the main source of household SHS exposure in 92% (*n* = 363) of the homes in which the mother was exposed; in the remaining 8% of houses, the source of exposure was another close family member. Exposure to SHS in public places was associated with maternal and paternal age (exposed mothers and husbands were younger, *P* = 0.01 and *P* < 0.001, respectively), ethnicity, and parity. Specifically, Greek women (66%) reported higher exposure than did immigrant women (46%, *P* < 0.001), as did women with Greek husbands (66%), as compared with those with immigrant husbands (48%, *P* = 0.002). Exposure to SHS in a car was reported by 28% of pregnant nonsmoking women (*n* = 231). Lower maternal and paternal education, as well as younger age, were associated with self-reported exposure (*P* < 0.001 in all cases), which was similar to the findings regarding self-reported exposure to SHS from other sources, such as social gatherings and visits. It is noteworthy that only 6% of women reported that they were not exposed to SHS from any source (household, work, public places, car, other sources) during the first 12 weeks of pregnancy; thus, 94% were exposed, and 44% of nonsmoking women reported being exposed to SHS from at least 3 different sources.

**Table 3.** Sources of secondhand smoke exposure by sociodemographic characteristics in nonsmoking^a^ pregnant women in the Greek RHEA birth cohort study, 2007–2008

		Exposure at home		Exposure at work^d^		Exposure in public place		Exposure in car		Exposure in other places	
						
		Exposed	Unexposed	Exposed	Unexposed	Exposed	Unexposed	Exposed	Unexposed	Exposed	Unexposed
Age^b^	*Maternal age (m ± SD)*	29.3 ± 5.2*	30.7 ± 4.4	30.2 ± 4.0*	31.7 ± 4.6	29.3 ± 4.9*	30.3 ± 5.2	28.0 ± 5.3*	30.3 ± 4.8	28.9 ± 5.0*	30.8 ± 4.9
	*Paternal age (m ± SD)*	33.2 ± 5.7*	34.3 ± 5.7	33.1 ± 5.0*	35.1 ± 5.5	32.9 ± 5.7*	34.4 ± 5.8	32.2 ± 5.9*	34.0 ± 5.6	32.9 ± 5.6*	34.4 ± 5.9

Residence^c^	*Urban %*	71	28.8	51.1	48.9	64.7	35.3	25.0*	75.0	57.3*	42.7
	*Rural %*	75	25.0	37.5	62.5	60.4	39.6	37.3*	62.7	66.3*	33.7

Ethnicity^c^	*Greek %*	72	27.9	48.9	51.1	66.2*	33.8	27.8	72.2	59.9	40.1
	*Non-Greek %*	70	30.5	48.6	51.4	46.3*	53.7	28.6	71.4	57.1	42.9

Paternal ethnicity^c^	*Greek %*	72	27.7	49.3	50.7	65.6*	34.4	27.6	72.4	59.6	40.4
	*Non-Greek %*	68	31.8	43.8	56.3	48.2*	51.8	30.7	69.3	59.1	40.9

Education^c^	*Low %*	84*	15.7	43.2	56.8	58.8	41.2	39.3*	60.7	66.1*	33.9
	*Medium %*	75*	24.7	53.0	47.0	64.0	36.0	31.6*	68.4	63.9*	36.1
	*High %*	58*	41.9	53.8	53.8	67.1	32.9	15.6*	84.4	48.9*	51.1

Paternal education^c^	*Low %*	84	16.4	49.4	50.6	62.3	37.7	42.9*	57.1	72.1*	27.9
	*Medium %*	74*	25.6	50.3	49.7	63.8	36.2	26.0*	74.0	61.7*	38.3
	*High %*	48*	52.2	52.8	52.8	66.1	33.9	9.2*	90.8	38.0*	62.0

Paternal smoking status	*Nonsmoker %*	50*	50.3	50.2	49.8	63.3	36.7	12.3*	87.7	55.3*	44.7
	*Smoker %*	100*	0.0	46.9	53.1	64.5	35.5	47.9*	52.1	65.0*	35.0

Parity^c^	*Multipara %*	71	29.2	46.1	53.9	61.1^†^	38.9	27.5	72.5	58.1	41.9
	*Primipara %*	74	26.5	53.5	46.5	68.4^†^	31.6	28.6	71.4	61.9	38.1

No. of children in house^c^	*1–2*	67*	33.1	48.9	51.1	64.3	35.7	25.7	74.3	58.3	41.7
	*3+*	79*	20.9	45.3	54.7	56.4	43.6	32.3	67.7	58.7	41.3

Total population % (*n*)		71.8 (584)	28.2 (229)	48.8 (189)	51.2 (198)	63.8 (515)	36.2 (292)	27.9 (231)	72.1 (597)	40.5 (493)	59.5 (335)

^a^Nonsmoking status was defined as a pregnant woman who had not smoked cigarettes for at least 3 months before conception; *P* values based on 2-sided tests: ^†^<0.05, *<0.001.

^b^2-sided *t*-test.

^c^2-sided chi-square test.

^d^387 women worked during pregnancy; only these women were analyzed.

Factors other than paternal smoking that were independently associated with exposure to SHS during pregnancy, after controlling for possible confounders, are shown in Table 4. Household exposure to SHS was mediated by paternal and maternal education, and by paternal ethnicity. As compared with women with higher-educated husbands, women with lower-educated husbands were more likely to be exposed to SHS in the house (OR, 2.87; 95% CI, 1.99–4.15; *P* < 0.001), car (2.88; 1.63–5.06; *P* < 0.001), other places (3.53; 2.53–4.92; *P* < 0.001), and from more sources (2.54; 1.67–3.86; *P* < 0.001). Lower maternal education was also related to higher exposure in the car (OR, 1.54) and to more sources of exposure (1.50). Maternal age was an important factor that mediated SHS exposure, as elder mothers were less likely to be exposed to SHS (OR, 0.92–0.98 per 1 year increase). Furthermore, maternal exposure to SHS in public venues was only associated with maternal age (older mothers were more likely to be exposed; OR, 1.02 per 1 year increase) and ethnicity (non-Greek mothers were less likely to be exposed than their Greek peers; OR, 0.44). Finally, when the number of sources of exposure to SHS was taken into account, parity was a significant mediator in addition to those noted above, as multiparous women were less likely to be exposed to SHS than were women who had no other children (OR, 0.69; 95% CI, 0.50–0.96; *P* = 0.028).

**Table 4.** Factors associated with exposure to secondhand smoke among nonsmoking pregnant women in the Greek RHEA birth cohort study, 2007–2008

		OR^a^	95% CI	*P*-value^b^
**Exposure to SHS in house**				
Paternal ethnicity	Greek	1.00		0.017
	Non-Greek	0.49	0.28–0.88	
Paternal education	High	1.00		<0.001
	Mid/low	2.87	1.99–4.15	

**Exposure to SHS in public venue**				
Maternal ethnicity	Greek	1.00		
	Non-Greek	0.44	0.27–0.70	0.001
Age	Change per year	1.02	1.01–1.03	<0.001

**Exposure to SHS in car**				
Age	Change per year	0.92	0.91–0.94	<0.001
Paternal education	High	1.00		
	Mid/low	2.88	1.63–5.06	<0.001
Maternal education	High	1.00		
	Mid/low	1.54	0.98–2.42	0.060
Residence	Urban	1.00		
	Rural	1.54	1.05–2.24	0.026

**Exposure to SHS in other places**				
Age	Change per year	0.98	0.97–0.99	<0.001
Paternal education	High	1.00		
	Mid-Low	3.53	2.53–4.92	<0.001

**Higher SHS exposure (>2 sources vs. 0–2 sources)**				
Age	Change per year	0.98	0.96–0.99	<0.001
Maternal ethnicity	Greek	1.00		
	Non-Greek	0.45	0.27–0.74	0.002
Educational level	High	1.00		
	Mid/low	1.50	1.03–2.18	0.034
Paternal education	High	1.00		
	Mid/low	2.54	1.67–3.86	<0.001
Parity	Primipara	1.00		
	Multipara	0.69	0.50–0.96	0.028

^a^Backward logistic regression analysis among nonsmokers was conducted with the following factors in step 1: maternal and paternal age, level of education (high vs. mid/low), ethnicity (Greek vs. immigrant), parity (primipara vs. multipara), and place of residence (urban vs. rural).

^b^*P* values based on 2-sided tests, with variables excluded when *P* > 0.1. The level of statistical significance was defined as *P* < 0.05.

## DISCUSSION

In the present study, 36% of pregnant women reported smoking some time during the first weeks of pregnancy, and half of them quit before week 12. Quitting was related primarily to their husband’s characteristics: women married to men who were Greek, higher-educated, or nonsmokers were more likely to quit than their respective peers. Among nonsmoking pregnant women, 94% reported as least some exposure to SHS during pregnancy, with the majority reporting SHS exposure in the home or in public places. Lower-educated women and women with lower-educated spouses were more likely to be exposed to SHS, as were younger women.

The percentage of pregnant women who reported smoking at the beginning of and before pregnancy was very similar to the prevalence of smoking among women in Greece. Previous research indicated that the prevalence of active smoking among women in Greece ranges from 23% to 39%, while population-based studies of parents of preschool children in Crete^18^ revealed a similar percentage, 38%. Maternal pre-pregnancy smoking status was independently related to the husband’s age, ethnicity, and smoking status, and to maternal education and place of residence. Among these factors, being married to a smoker was the strongest predictor of maternal smoking status, a fact that could be influenced by partner selection. These findings are similar to those noted in previous studies, which highlighted the role of educational inequalities and rural/urban differences on smoking prevalence among European adults.^19^^–^^21^

Smoking cessation is a critical aspect of harm reduction during pregnancy, and is dependent on a number of personal, family, educational, and social characteristics and beliefs such as age, awareness, education, occupation, social status, residence, parity, stress, partner smoking status, and even psychological factors such as fear of excessive weight gain during pregnancy.^12^^–^^17^^,^^22^^,^^23^ Although a large number of studies have identified maternal factors as strong predictors of spontaneous cessation during pregnancy, we found that, in Crete, paternal and family characteristics were a stronger indicator of maternal cessation, ie, those less likely to quit smoking during pregnancy were married to a smoker or to have more than 1 child. Indeed, evidence has suggested that the existence of past pregnancies which resulted in the birth of healthy children undermines the motivation to cease smoking during a subsequent pregnancy, which could be the case among the women of our study population.^24^ Moreover, neither maternal nor paternal education status was found to be statistically associated with smoking cessation after conception.

The detrimental effects of fetal exposure to SHS are well established. Despite the known adverse pregnancy outcomes and developmental effects of such exposure, almost all pregnant women were exposed, and the household was the main source of exposure. The avoidance of household SHS exposure is believed to be associated with strong social determinants, such as the existence of household smoking bans, the number of cigarettes smoked per day, parental educational status, and awareness of the harm of SHS.^25^^,^^26^ We, too, found that maternal and paternal educational status were strong determinants of household exposure to SHS, with the latter playing a more significant role. The effect of paternal ethnicity was confounded by education level since most non-Greek partners of nonsmoking women had a low education level. When education was evaluated in the multivariate model, the ethnicity of the husband was associated with exposure to SHS at home. Exposure to SHS in a car was also determined by paternal educational status and age, similar to the results of other studies in which the father’s educational status was a stronger predictor than maternal education of automobile SHS exposure.^27^ After controlling for possible confounders, lower-educated women of Greek origin, those married to lower-educated men, those who were older, and those with only 1 child were found to be exposed to SHS from a larger number of sources. Although this categorization does not take into account different times, frequencies, or levels of exposure, we hypothesize that mothers exposed to SHS from a larger number of sources have greater overall exposure to SHS than do women with fewer sources of exposure. Moreover, the second largest source of exposure among the women in our study population was exposure to SHS in public places, with older women and women of non-Greek origin less likely to be exposed—a fact that we attribute to social factors, as it is very common for young people, mainly of Greek ethnicity, to patronize public venues such as cafés and restaurants.

Research has documented elevated levels of SHS in the majority of public venues in Greece, despite the existence of a partial smoking ban^28^ that is flagrantly ignored.^29^ The significance of the application of and adherence to a smoking ban is apparent when one takes into account both immediate and long-term health benefits.^30^ Smoke-free policies can have a profound effect on the population’s level of exposure to SHS, and can be an effective strategy for reducing both active smoking and SHS exposure among pregnant women, as was seen after the implementation of a comprehensive smoking ban and educational campaign in neighboring Italy.^31^

The present study’s design and relatively large and representative sample allow us to interpret the results with confidence and permit generalizability among the population of Crete. However, it should be noted that the results on active smoking are based on self-reported data and are therefore subject to bias, as it is unclear whether these self-reported measures during pregnancy are underreported or accurate.^32^ If current smoking status was underreported, this would result in lower prevalences, which would make the results even stronger. Data on the number of cigarettes before pregnancy was not available; therefore, we were not able to analyze that covariate in the regression analysis. Additionally, exposure to SHS was not evaluated with the use of a biomarker (eg, cotinine), but such analysis is likely in the future. Despite this, women are more likely to have underreported their exposure to SHS; thus, their exposure may be even higher than noted in this report.^33^

Both active smoking and exposure to SHS are significant threats to public and prenatal health in Greece. Taking into account the elevated percentage of mothers that either continue to smoke during pregnancy or are exposed to SHS, the necessity of developing educational awareness programs is undeniable. Such educational campaigns, provided either during obstetrical visits or via a mass media intervention campaign, should focus on younger, less-educated, mothers and their spouses, who were identified in this study as more likely to be smokers before conception. Moreover, as more than a third of women reported smoking at some time during pregnancy, these educational interventions should also be addressed to both youths and women of reproductive age, so as to reduce tobacco use and educate the population regarding the ramifications of fetal exposure to tobacco constituents. Additionally, because a significant source of SHS exposure during pregnancy was exposure in public places, the enforcement of a nationwide comprehensive smoking ban in public places in Greece is imperative.
